# Psychometric properties of the Turkish version of the Preschool Eating, Lifestyle, and Sleeping Attitudes (PRELSA) scale

**DOI:** 10.1186/s12887-026-07532-9

**Published:** 2026-08-14

**Authors:** Edanur Tar Bolacali, Memiş Bolacali

**Affiliations:** 1 Faculty of Health Sciences, Department of Children Health and Disease Nursing, Kirsehir Ahi Evran University, Kirsehir, Türkiye; 2 Faculty of Medicine, Department of Biostatistics and Medical Informatics, Kirsehir Ahi Evran University, Kirsehir, Türkiye

**Keywords:** Childhood obesity, Parents, Preschool child, Psychometrics, Validation study

## Abstract

**Background:**

Childhood obesity in preschool years is strongly influenced by modifiable parental attitudes and behaviors. This study examined the validity and reliability of the Turkish version of the Preschool Eating, Lifestyle, and Sleeping Attitudes (PRELSA) Scale, which assesses parental attitudes related to childhood obesity during the preschool period.

**Methods:**

This methodological study was conducted with 460 parents of children aged 2–6 years from seven preschools in Central Anatolia, Türkiye. Data were collected using a family and child information form and the PRELSA Scale. The adaptation process included forward–backward translation, expert review, face validity assessment, and psychometric evaluation. Data were analyzed using exploratory and confirmatory factor analyses, Cronbach’s alpha, test–retest reliability, and intraclass correlation coefficients (*ICC*).

**Results:**

The Turkish version comprised 39 items across 13 subscales. The overall Cronbach’s alpha coefficient was 0.887. Exploratory factor analysis explained 58.5% of the total variance, with factor loadings ranging from 0.353 to 0.890. Confirmatory factor analysis indicated acceptable model fit (*χ²/df* = 1.434, *RMSEA* = 0.031, *SRMR* = 0.046, *CFI* = 0.904, *IFI* = 0.907, *GFI* = 0.911, *TLI* = 0.888). Test–retest reliability was high (*r* = 0.911), and the ICC was 0.865 (95% CI: 0.738–0.933), indicating good temporal stability.

**Conclusion:**

The Turkish PRELSA Scale is a valid and reliable instrument for assessing parental attitudes toward eating behaviors, physical activity, screen use, and sleep routines in early childhood.

**Supplementary Information:**

The online version contains supplementary material available at https://doi.org/10.1186/s12887-026-07532-9.

## Introduction

Childhood obesity is defined as the child consuming more calories than they expend, leading to an increase in body fat, and having a body mass index percentile above the 95th percentile when compared to age and gender-specific percentile curves [1, 2]. Data reported by the World Health Organization (WHO) indicate that in 2022, over 390 million children and adolescents aged 5–19 were classified as overweight, and approximately 160 million of these individuals were living with obesity. In 1990, the prevalence of obesity among children and adolescents aged 5–19 was 2% (31 million young people), while this rate is reported to have risen to 8% (more than 160 million young people) in 2022 [3,4]. In the preschool period, an estimated 35 million children under the age of 5 were overweight in 2024 [4]. Studies conducted in Türkiye also indicate that the prevalence of childhood obesity follows a similar pattern and that this condition lays the foundation for lifelong health problems [5, 6]. Childhood obesity, which is becoming an increasingly prevalent public health issue today, causes many physical, psychological, and social problems during childhood [7] and also paves the way for obesity and chronic diseases in adulthood [8]. Considering the negative consequences of childhood obesity, the importance of combating obesity from early childhood becomes apparent [7, 8].

There are three critical periods in the development of childhood obesity: the first thousand days (pregnancy and the first two years of life), the adipose rebound period (an increase in body mass index occurring around age 6), and adolescence. Among these critical periods, the preschool years are particularly important for acquiring and maintaining healthy habits. Lifestyle habits acquired during this period, such as nutrition, physical activity, screen time, and sleep patterns, significantly affect the child’s future health [1, 2, 7–9]. Most risk factors associated with obesity in the preschool period are considered to be significantly modifiable factors [2, 5, 10]. In childhood obesity, it has been reported that parental attitudes and behaviors are also effective, along with genetic, environmental, and psychosocial factors, and parents play a very important role in combating obesity [9, 11]. Parents play an active role in developing their children’s healthy lifestyle behaviors, such as “eating habits, physical activity, and sleep patterns,” starting from early childhood. Therefore, in order to combat childhood obesity, it is necessary to work in collaboration with parents to develop these behaviors, which are among the protective factors, and to ensure that they are supported by health professionals [9, 11]. Furthermore, it is thought that examining the relationship between childhood obesity and parents’ attitudes and behaviors regarding healthy lifestyles will contribute to the identification of obesogenic risk factors [12]. Various studies in the literature have examined the interaction between parental attitudes and childhood obesity risk however, these studies generally focus on specific behaviors such as diet or physical activity [1, 2, 9, 13].

Reliable and valid psychometric instruments are needed to assess parental attitudes and behaviors related to childhood obesity. The current literature contains various scales that measure different dimensions such as parents’ feeding practices, attitudes toward physical activity, and sleep habits however, these do not cover all behavioral dimensions related to obesity [10, 14, 15]. In a study conducted by Carretero-Bravo et al., the PRELSA Scale, which comprehensively examines parental attitudes and behaviors associated with childhood obesity, was developed; the scale was subsequently revised and finalized in a later study [16]. The PRELSA Scale offers a comprehensive approach to assessing risk factors for childhood obesity by covering various dimensions of nutrition, lifestyle, and sleep habits [12, 16]. A review of the literature reveals that, despite the existence of some measurement tools examining parental attitudes and behaviors toward childhood obesity in the Turkish population, it is evident that there are limited valid and reliable measurement tools that assess parental attitudes related to childhood obesity in the preschool period in a multidimensional manner [6, 17, 18]. There is a need for a culturally adapted and psychometrically tested scale that comprehensively assesses the obesogenic attitudes of parents of preschool-aged children in Türkiye. Based on this, the aim of this study is to adapt the PRELSA Scale into Turkish, ensure its linguistic equivalence, and examine its validity and reliability in Turkish culture. It is believed that this scale will be an important tool for both researchers and health and education institutions in Turkish society in developing interventions aimed at preventing childhood obesity.

## Methods

### Design

This research was conducted as a methodological study to establish the validity and reliability of the PRELSA scale.

### Setting and sample

The study was carried out through face-to-face data collection with parents of children aged 2–6 years who were enrolled in seven kindergartens located in the Central Anatolia region of Türkiye between February and June 2025. Eligible participants were Turkish parents who had a preschool child within this age range, served as the child’s primary caregiver (mother or father), were able to read and comprehend Turkish, and provided voluntary informed consent to participate. In methodological guidelines for scale development studies, it is generally recommended that the sample size include approximately 5 to 10 participants per item [19]. At least 240 participants were required to determine the psychometric properties of the Turkish version of the 48-item PRELSA Scale. According to the International Test Commission, factor analysis in psychometric research requires a sample size of no fewer than 200 participants. Following this guideline, data were collected from 460 parents, excluding the 30 participants involved in the preliminary pilot study [20].

### Data collection tools

#### Family and child information form

The Information Form was prepared by researchers in line with the literature and consists of ten questions aimed at determining parental age, parental status, parental education level, employment status, place of residence, income status, number of children, child’s age, child’s birth order, and child’s gender [5, 16].

#### Preschool Eating, Lifestyle, and Sleeping Attitudes (PRELSA) scale

The PRELSA Scale was developed by Carretero-Bravo et al. [12], to assess the attitudes of parents with preschool-aged children (2–6 years old) toward nutrition, lifestyle, and sleep related to childhood obesity. The scale addresses these three key areas, which are among the important risk factors for childhood obesity, in the context of parental attitudes. The psychometric properties of the PRELSA Scale were subsequently re-evaluated by Carretero-Bravo et al. [16], using an expanded sample; it was reported that the scale’s structure, consisting of a total of 48 items and 14 subscales, is valid and reliable. The scale consists of the following sub-dimensions: Child’s Weight Concern (CWC) (1, 2), Use of Rewards (REW) (3, 4, 6), Pressure to eat (PRE) (7, 8, 9, 10, 11), Variety and Quality of the Diet (VQD) (12, 13, 14, 15, 16, 17), Movement during Mealtime (MOV) (18, 19, 20), Structured Mealtime (SMT) (21, 22, 23), Family Meal Environment (FME) (24, 25), Distractions during Mealtime (DIS) (26, 27, 28), Importance of Physical Activity (IPA) (29, 30, 31), Support and Co-Participation in Physical Activity (SCP) (32, 33, 34, 35, 36, 37, 38, 39), Motivation to Physical Activity (MOT) (40, 41), Limits Screen Viewing (LSV) (42, 43), Impact and Modification of Sleep (IMS) (44, 45, 46), Importance of Routines regarding Sleep (IRS) (47,48). The scale is a 5-point Likert scale, and responses to the first subscale, “Child’s Weight Concern,” are rated as follows: “1 = Not worried at all, 2 = A little worried, 3 = Worried, 4 = Quite worried, 5 = Very worried.” For all other questions, the responses are “1 = Disagree, 2 = Slightly disagree, 3 = Indifferent/don’t care, 4 = Slightly agree, 5 = Agree,” and parents are asked to indicate how much each statement applies to them. The scale is scored by calculating the average score for each sub-dimension and the total average score covering all sub-dimensions. Parents who scored high on this scale were defined as “parents who demonstrate appropriate attitudes and behaviors aimed at preventing obesity.” Therefore, an increase in the total score obtained from the scale indicates that parents have lower levels of risky attitudes associated with childhood obesity. Items 1, 2, 3, 4, 5, 6, 7, 9, 11, 14, 18, 20, 26, 27, and 28 of the scale are reverse-coded items. Cronbach’s Alpha values range from 0.72 to 0.97 for the different dimensions of the scale. The Cronbach’s Alpha value was calculated as 0.89 across the entire scale [16]. The Turkish version of the PRELSA Scale demonstrated a Cronbach’s alpha coefficient of 0.887.

### Data collection

Data collection began after ethical approval, institutional authorization, and permission to adapt the PRELSA Scale were obtained. The study was conducted in seven randomly selected kindergartens. School administrators and teachers were informed about the study procedures prior to implementation. Eligible parents of children aged 2–6 years were identified through teachers. Informed consent forms were distributed via children’s school bags, and additional study information was shared through class WhatsApp groups. Parents who provided written consent were invited to the school and completed a sociodemographic form and the PRELSA Scale in a quiet classroom using a self-report method. To assess test–retest reliability, participants created a confidential unique code during the first administration. Four weeks later, 30 parents completed the scale again using the same code. Data collection was scheduled at convenient times to minimize respondent burden. All procedures were conducted in accordance with confidentiality and anonymity principles.

### Translation process

During linguistic validation, the PRELSA Scale was translated into Turkish by a bilingual academic in collaboration with a professional translator. The Turkish version was independently back-translated into English by two academics and compared with the original to confirm conceptual equivalence. Clarity and comprehensibility were evaluated in a pilot test with 30 parents, and minor revisions were made based on feedback and research team consensus. The final Turkish version was established following established cross-cultural adaptation guidelines [21].

### Experts’ opinions

To assess content validity, the Turkish version of the PRELSA Scale was evaluated by ten independent specialists from the fields of pediatric nursing and nutrition and dietetics. These experts were academics with professional experience in childhood obesity research and practice. Following the Davis technique, they assessed the scale at both the item level (I-CVI) and the scale level (S-CVI).

### Face validity

Face validity was assessed by five parents of children aged 2–6 years, who evaluated item clarity, comprehensibility, and relevance. Feedback indicated that the overall structure was appropriate; however, minor linguistic revisions were made to simplify wording and combine similar statements. These modifications aimed to enhance clarity and ensure that the items accurately reflected the intended constructs for the target population.

### Preliminary test

A pilot study was conducted with 30 eligible parents who were not included in the main sample. The items were found to be understandable, and the scale was finalized. The final version was then administered face-to-face to the main sample.

### Data analysis

**Fig. 1:**
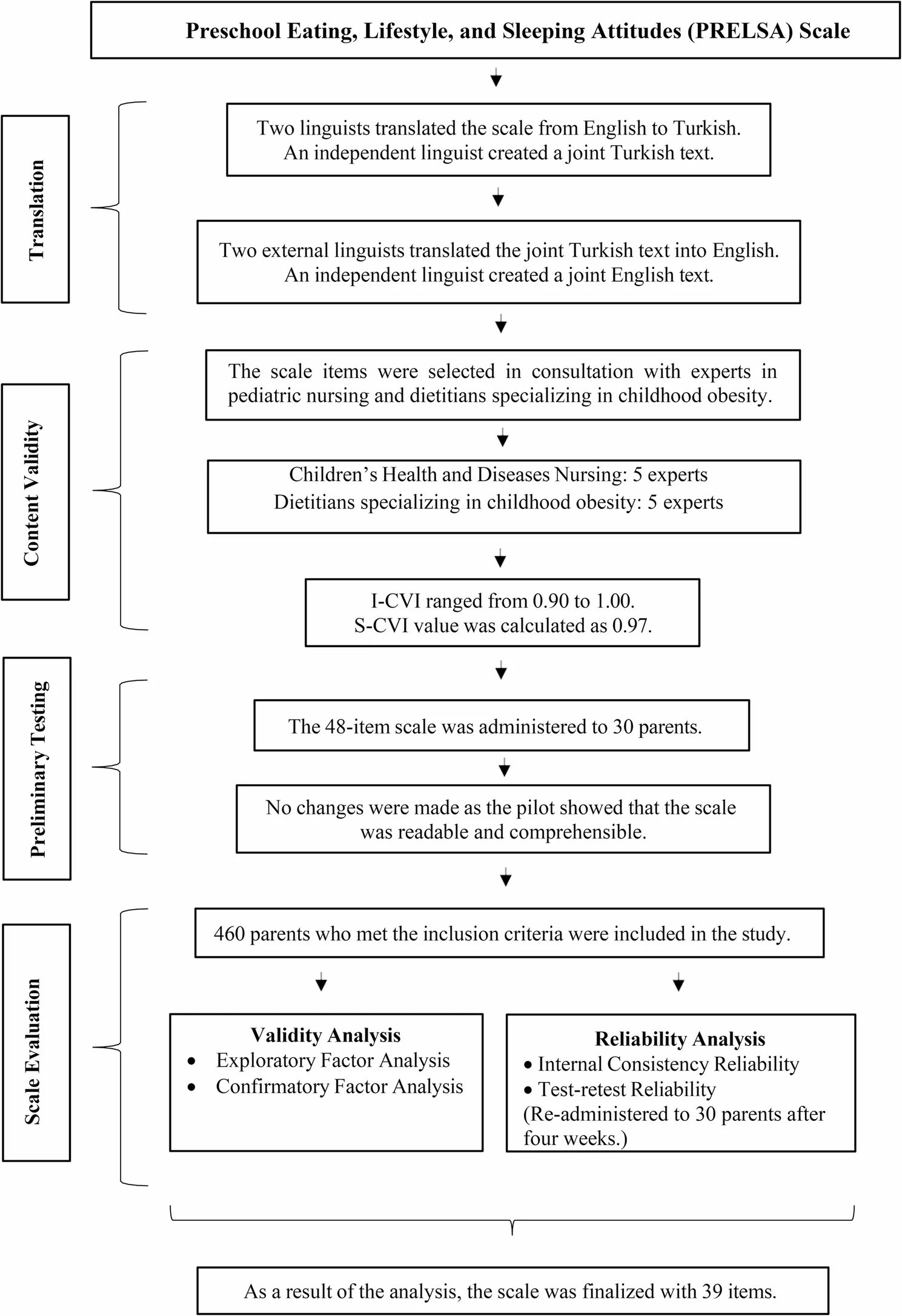
Research process

Statistical analyses were performed using IBM SPSS version 26.0 and AMOS version 23.0. Descriptive measures such as means, standard deviations, frequencies, and percentages were computed. The construct validity of the Turkish PRELSA Scale was examined through Exploratory Factor Analysis (EFA) and Confirmatory Factor Analysis (CFA) based on data collected from parents. First, CFA was performed to test whether the original 48-item, 14-factor structure was confirmed in the Turkish sample. Since the original model was not adequately supported, EFA was subsequently conducted. Then, CFA was used to test the factor structure obtained from EFA. For validity and reliability assessment, the Kaiser–Meyer–Olkin (KMO) measure, Bartlett’s test of sphericity, and various goodness-of-fit indicators were applied. Within the CFA framework, model adequacy was evaluated using the chi-square to degrees of freedom ratio (χ²/df), Root Mean Square Error of Approximation (RMSEA), Standardized Root Mean Square Residual (SRMR), Comparative Fit Index (CFI), Incremental Fit Index (IFI), Goodness-of-Fit Index (GFI), and Tucker–Lewis Index (TLI). Reliability was assessed using Cronbach’s alpha, McDonald’s omega, item–total correlations, the Intraclass Correlation Coefficient (ICC), and test–retest analysis. Statistical significance was set at *p* < 0.05 with a 95% confidence interval. The Turkish adaptation process included forward–backward translation, expert review, pilot testing, and psychometric evaluation. The adaptation and validation process is shown in Fig. 1.

## Results

### Participant characteristics

A total of 460 parents were included in this study. The average age of the participating parents was 35.20 ± 5.47 years, and the average number of children was determined to be 2.20 ± 0.86. Mothers constituted the vast majority of participants (86.1%). When the educational level of the parents was examined, the highest rate was found among associate degree and bachelor’s degree graduates (43.3%). It was determined that most of the participants were not working (57.8%), that the majority of families lived in the city center (77.8%), and that the most common income level reported was income equal to expenditure (63.9%). In terms of the gender distribution of children, it was found that the proportion of boys was higher (51.7%) (Table 1).

**Table 1:** Sociodemographic characteristics of parents and child (*n* = 460)

Characteristics		Mean ± SD (min- max)	
Parent age		35.20 ± 5.47 (21–53)	
Number of children		2.20 ± 0.86 (1–5)	
Child’s age		5.41 ± 0.62 (2–6)	
Child’s birth order		1.87 ± 0.93 (1–5)	

		Frequency (*n*)	Percentage (%)
Parental status	Mother	396	86.1
	Father	64	13.9
Parent education level	Primary school	40	8.7
	Secondary school	52	11.3
	High school	168	36.7
	Associate and Bachelor’s Degree	199	43.3
Work status	Full-time work	156	33.9
	Part-time work	38	8.3
	Not working	266	57.8
The place where the family lives	City center	358	77.8
	District	102	22.2
Perceived income level	Income exceeds expenses	85	18.5
	Income equals expenses	294	63.9
	Income is less than expenses	81	17.6
Child gender	Girl	222	48.3
	Boy	238	51.7

*SD* Standard Deviation, *min* Minimum, *max* Maximum

### Validity analysis

#### Content validity

The draft form of the scale was evaluated by a total of 10 academics specializing in pediatric nursing (*n* = 5) and nutrition and dietetics (*n* = 5). The Davis technique was used to evaluate the scale items. As a result of the evaluation, it was determined that the content validity index (I-CVI) at the item level ranged from 0.90 to 1.00, while the content validity index (S-CVI) at the scale level was 0.97. These results indicate a high level of agreement among experts [19, 22].

#### Construct validity

The original 48-item, 14-factor structure proposed by Carretero-Bravo et al. was initially tested using CFA. Although some fit indices were acceptable (χ²/df = 1.54, RMSEA = 0.034, GFI = 0.881, AGFI = 0.859), the model showed inadequate comparative fit indices (CFI = 0.837, TLI = 0.814) and produced inadmissible parameter estimates, including convergence problems, a negative error variance, and standardized factor loadings exceeding 1.0. Therefore, the original structure was not adequately supported in the Turkish sample. Subsequently, EFA was conducted to determine the most appropriate factor structure.

#### Explanatory Factor Analysis (EFA)

EFA was conducted to examine the construct validity of the scale. The Kaiser–Meyer–Olkin (KMO) coefficient was found to be 0.719, and Bartlett’s test of sphericity yielded significant results (*χ²* = 3505.367, *p* < 0.001). These findings indicated that the dataset was suitable for factor analysis. As a result of the EFA, the factor loadings of the 48-item PRELSA Scale were examined, and six items (6, 10, 12, 13, 14, and 37) with factor loadings below 0.30 were removed from the scale on the grounds that they did not adequately represent the relevant factor. In addition, three items (8, 23, and 24) with cross-loading differences below 0.10 and similar loadings on more than one factor were considered problematic and excluded from the analysis. Following these modifications, the scale reached a final structure consisting of 39 items. The repeated EFA yielded a 13-factor structure with eigenvalues greater than 1, and these factors were found to explain 58.5% of the total variance. The factor loadings of the items in the final structure ranged from 0.353 to 0.890 (Table 2).

**Table 2:** Factor loadings from the exploratory factor analysis

	Items	Item Content	Loading	Eigenvalues	Explained variance (%)
Factor 1	Item 30	I consider my child’s physical activity as important for their development as their studies.	0.798	4.852	12.441
	Item 29	I consider my child’s physical activity as important for their development as their nutrition.	0.725		
	Item 31	I try to teach my child that being active is good for their health.	0.701		
	Item 38	I believe it’s necessary to have time for physical activity with my child.	0.602		
	Item 39	I believe family habits of physical activity can influence my child’s healthy lifestyle throughout their life.	0.478		
Factor 2	Item 26	I consider it appropriate for my child to eat entertained with screens (TV, computer, mobiles, and/or tablets).	0.825	2.494	6.394
	Item 28	I consider it appropriate to use screens during meals.	0.808		
	Item 27	I consider it appropriate for my child to eat entertained with some game.	0.644		
Factor 3	Item 35	I consider it necessary to organize my child’s routines so that there is frequently space for physical activity.	0.668	1.855	4.757
	Item 34	I consider it necessary to provide my child with toys and/or games that encourage physical activity.	0.622		
	Item 33	I believe my child can learn healthy habits of physical activity through my example.	0.605		
	Item 32	I consider it important to introduce my child to various options for physical activity and active play.	0.353		
	Item 36	One of my priorities when thinking about where to live is that my child has spaces to play indoors or outdoors.	0.397		
Factor 4	Item 48	I consider it appropriate for my child to sleep most nights in the same place.	0.746	1.790	4.590
	Item 47	I consider it appropriate for my child to have the same sleep habits every day of the week.	0.703		
	Item 46	I believe family routines can influence proper sleep patterns for children.	0.630		
Factor 5	Item 2	Are you concerned that potential excess weight in your child during this preschool stage could be a health problem in the short/medium term?	0.890	1.629	4.176
	Item 1	Are you worried that your child eats too much?	0.863		
Factor 6	Item 4	I consider it appropriate to use food to calm my child down.	0.718	1.493	3.829
	Item 5	I consider it appropriate to offer rewards to my child to keep them still.	0.704		
	Item 3	I consider it appropriate to use some type of food as a reward when behaving well.	0.541		
Factor 7	Item 41	I consider it appropriate to play active games and/or sports with my child over other activities.	0.836	1.413	3.623
	Item 40	I consider it appropriate to motivate my child to engage in physical activities over other types of activities.	0.829		
Factor 8	Item 21	I consider it appropriate for my child to be able to decide when he/she wants to eat.	0.808	1.321	3.387
	Item 22	I consider it appropriate for my child to sometimes eat between routine meals.	0.774		
Factor 9	Item 45	I believe my child’s lack of sleep can encourage them to eat more during the day.	0.867	1.273	3.264
	Item 43	I consider it appropriate to limit my screen time when I’m with my child/children.	0.719		
	Item 42	I consider it appropriate to limit my child’s screen viewing (TV, computer, mobiles, and/or tablets).	0.680		
	Item 44	I believe sleep routines affect other areas of my child’s mental and physical development.	0.567		
Factor 10	Item 17	I consider it necessary for family meals to always have dishes with salads and/or vegetables on the table.	0.717	1.222	3.134
	Item 15	I consider it necessary to always offer salads and/or vegetables in my child’s meals.	0.661		
	Item 16	I consider it necessary for my child to eat fruits every day.	0.637		
Factor 11	Item 18	I consider it appropriate for my child to get up or move around during meals.	0.738	1.218	3.123
	Item 19	I consider it appropriate for my child to remain seated throughout mealtime.	0.738		
	Item 20	I consider it appropriate for my child to eat if they are not seated at the table.	0.513		
Factor 12	Item 9	I try to make my child eat regardless of whether they say they are not hungry.	0.729	1.138	2.919
	Item 7	I believe my child should always finish all the food on the plate.	0.659		
Factor 13	Item 11	I consider it necessary for my child to eat enough, even if the diet is sometimes not varied.	0.717	1.120	2.872
	Item 25	I consider it necessary for my child to eat the same food as the rest of the family.	0.673		
Explained total variance (%)		58.509			
KMO coefficient		0.719			
Bartlett test		3505.367 (*p* < 0.001)			

As a result of factor analysis, the sub-dimensions of “Parental Concern about the Child’s Weight (CWC) (1, 2), Use of Food as a Reward (REW) (3, 4, 5), Pressure to Eat (PRE) (7, 9), Variety and Quality of the Diet (VQD) (15, 16, 17), Movement during Mealtime (MOV) (18, 19, 20), Structured Mealtime (SMT) (21, 22), Use of Distractions during Mealtime (DIS) (26, 27, 28), Parental Support and Co-Participation in Physical Activity (SCP) (32, 33, 34, 35, 36), Motivation to Physical Activity (MOT) (40, 41)” have been preserved. In addition, Item 11 in the Pressure to Eat (PRE) subscale and Item 25 in the Family Meal Environment (FME) subscale loaded together to form a new factor, which was named “Family-Based Feeding Expectations (FBFE) (11, 25)”. The sub-dimension “Importance of Physical Activity” has been amended by adding Items 38 and 39 from the sub-dimension “Support and Co-Participation in Physical Activity (SCP).” The factor name remains unchanged as “Importance of Physical Activity (IPA) (29, 30, 31, 38, 39).” Additionally, Items 44 and 45 belonging to the Impact and Modification of Sleep (IMS) subscale have been loaded onto the Limits Screen Viewing (LSV) subscale, while Item 46 has been loaded onto the Importance of Routines regarding Sleep (IRS) subscale. Accordingly, the factor name “Importance of Routines regarding Sleep (IRS) (46, 47, 48)” has been retained, while the other factor has been renamed “Attitudes Toward Screen Use and Sleep Patterns (ATSP) (42, 43, 44, 45)”. After item removal, reverse coding was applied to the remaining relevant items. Items numbered 1, 2, 3, 4, 5, 7, 9, 11, 18, 20, 26, 27, and 28 on the scale were reverse-coded for analysis. Preschool Eating, Lifestyle, and Sleeping Attitudes (PRELSA) Scale is provided as a supplementary file (Supplementary File 1).

#### Confirmatory Factor Analysis (CFA)

Following the EFA, CFA was conducted to verify the multidimensional structure of the PRELSA Scale consisting of 39 items and 13 subscales, and the initial model fit indices are presented in Table 3. After the initial analysis, theoretically meaningful modifications were made by correlating the error covariances between items 29 and 30, 30 and 31, and 34 and 35 in order to improve model fit. Following these modifications, the fit indices were found to fall within acceptable and good fit thresholds (χ²/df = 1.434, RMSEA = 0.031, SRMR = 0.046, CFI = 0.904, IFI = 0.907, GFI = 0.911, TLI = 0.888). These results confirm the multi-factor structure of the PRELSA Scale.

**Table 3:** Goodness-of-fit indices for the confirmatory factor analysis

Models/Data-model fit indices	Original 48-item 14-factor	Turkish 39-item 13-factor Before modification	Turkish 39-item 13-factor After modification
*χ* ^*2*^ */df*	1.542	1.632	1.434
*RMSEA*	0.034	0.037	0.031
*SRMR*	0.054	0.049	0.046
*CFI*	0.837	0.858	0.904
*IFI*	0.843	0.862	0.907
*GFI*	0.881	0.898	0.911
*TLI*	0.814	0.837	0.888

*χ2* Chi-square, *df* Degrees of Freedom, *RMSEA* Root Mean Square Error of Approximation, *SRMR* Standardized Root Mean Square Residual, *CFI* Comparative Fit Index, *IFI* Incremental Fit Index, *GFI* Goodness of Fit, *TLI* Tucker-Lewis Index

### Reliability analysis

#### Internal consistency reliability

Descriptive statistics and internal consistency analyses for the subscales of the scale are presented in Table 4. The number of items per factor ranged from 2 to 5, and item means ranged from 1.32 to 4.85. The total mean scores for the factors ranged from 2.64 to 24.25. Cronbach’s alpha coefficients for the PRELSA Scale subscales ranged from 0.714 to 0.928, while McDonald’s omega coefficients ranged from 0.716 to 0.938. For the total scale, Cronbach’s alpha and McDonald’s omega were 0.887 and 0.918, respectively. These results provide strong evidence for the internal consistency reliability of both the PRELSA Scale and its subscales (Table 4).

**Table 4:** Descriptive statistics and internal consistency of the PRELSA scale dimensions

Factor	Items		Range	Mean Item	Sum Mean	Cronbach’s Alpha	McDonald’s ω
*F1*: Importance of Physical Activity (IPA)		5	5–25	4.85 (0.27)	24.25 (1.39)	0.909	0.912
*F2*: Use of Distractions during Mealtime (DIS)		3	3–15	4.52 (0.68)	13.57 (2.05)	0.902	0.907
*F3*: Parental Support and Co-Participation in Physical Activity (SCP)		5	5–25	4.65 (0.36)	23.29 (1.83)	0.877	0.903
*F4*: Importance of Routines regarding Sleep (IRS)		3	3–15	4.76 (0.38)	14.30 (1.16)	0.888	0.889
*F5*: Parental Concern about the Child’s Weight (CWC)		2	2–10	1.32 (0.68)	2.64 (1.37)	0.928	0.938
*F6*: Use of Food as a Reward (REW)		3	3–15	4.16 (0.59)	12.48 (1.79)	0.806	0.812
*F7*: Motivation to Physical Activity (MOT)		2	2–10	4.59 (0.46)	9.19 (0.93)	0.896	0.896
*F8*: Structured Mealtime (SMT)		2	2–10	3.64 (0.97)	7.29 (1.94)	0.854	0.855
*F9*: Attitudes Toward Screen Use and Sleep Patterns (ATSP)		4	4–20	4.58 (0.32)	18.35 (1.28)	0.805	0.817
*F10*: Variety and Quality of the Diet (VQD)		3	3–15	4.69 (0.39)	14.08 (1.19)	0.791	0.847
*F11*: Movement during Mealtime (MOV)		3	3–15	4.40 (0.67)	13.21 (2.02)	0.771	0.836
*F12*: Pressure to Eat (PRE)		2	2–10	4.54 (0.55)	9.08 (1.11)	0.747	0.747
*F13*: Family-Based Feeding Expectations (FBFE)		2	2–10	4.25 (0.56)	8.51 (1.12)	0.714	0.716
Total PRELSA		39	39–195	4.36 (0.19)	170.30 (7.61)	0.887	0.918

*F *Factor

#### Inter-subscale correlations

The correlations between the 13 subscales of the PRELSA Scale are presented in Table 5. Positive and significant correlations were observed among the subfactors, with correlation coefficients ranging from 0.194 to 0.610 (*p* < 0.05). These results indicate a consistent and independent structure among the subscales (Table 5).

**Table 5:** Correlations among subscales and test–retest reliability of the PRELSA scale with 13 dimensions

Items	F1: IPA	F2: DIS	F3: SCP	F4: IRS	F 5: CWC	F 6: REW	F7: MOT	F8: SMT	F9: ATSP	F10: VQD	F11: MOV	F12: PRE	F13: FBFE	Test-Retest (*r*) (*n* = 30)
F1: IPA	1													0.749
F2: DIS	0.107*	1												0.873
F3: SCP	0.555**	0.125*	1											0.759
F4: IRS	0.348**	0.156*	0.394**	1										0.791
F5: CWC	-0.109*	-0.061	-0.112*	-0.088	1									0.730
F6: REW	0.083	0.340**	-0.082	-0.108*	-0.115*	1								0.895
F7: MOT	0.232**	0.082	0.318**	0.182**	0.081	-0.083	1							0.798
F8: SMT	-0.055	0.164**	0.052	-0.078	0.066	0.142*	-0.059	1						0.902
F9: ATSP	0.238**	0.059	0.257**	0.207**	0.099*	0.056	0.483**	-0.068	1					0.836
F10: VQD	0.272**	0.199**	0.284**	0.239**	-0.085	0.089	0.183**	-0.121*	0.138*	1				0.755
F11: MOV	0.153*	0.214**	0.167**	0.142*	-0.155*	0.192**	0.082	0.220**	0.089	0.098*	1			0.841
F12: PRE	0.245**	0.132*	0.204**	0.208**	-0.113*	0.105*	0.119*	0.054	0.080	0.382**	0.075	1		0.787
F13: FBFE	0.137*	0.069	0.135*	0.176**	-0.103	0.053	0.102*	0.059	0.061	0.085	0.143*	0.097	1	0.869
Total PRELSA	0.551**	0.548**	0.610**	0.483**	0.194**	0.415**	0.416**	0.387**	0.418**	0.451**	0.514**	0.397**	0.347**	0.911

*F *Factor,* *p < 0.05*,** *p < 0.01*

#### Test–retest reliability

Test–retest analysis (*n* = 30) results revealed that the scale demonstrated high stability over time. Reliability coefficients for the subscales ranged from 0.730 to 0.902, while the test–retest reliability for the total scale was found to be 0.911. The ICC was 0.865 (95% CI: 0.738–0.933), indicating good temporal stability of the Turkish version of the PRELSA Scale. This finding is consistent with the high test–retest correlation (*r* = 0.911, *p* < 0.001), together providing strong evidence for the temporal reliability of the scale. These findings support the PRELSA Scale as a reliable and consistent measurement tool.

#### Criterion-related validity

To provide additional evidence regarding criterion-related validity, correlations between PRELSA total scores and BMI-related variables were examined. PRELSA total scores demonstrated significant negative correlations with parental BMI (*r* = -0.414, *p* < 0.001) and child BMI (*r* = -0.355, *p* < 0.001). Given that higher PRELSA scores indicate more favorable parental attitudes and behaviors toward the prevention of childhood obesity, these findings support the expected relationship between PRELSA scores and obesity-related indicators.

## Discussion

Parental attitudes toward nutrition, physical activity, screen time, and sleep during the preschool years play a decisive role in the development of children’s healthy lifestyle behaviors. Evaluating these attitudes using valid and reliable measurement tools is of great importance in strengthening evidence-based practices for early childhood [5, 10, 14, 15]. The PRELSA Scale, adapted into Turkish for this study, is a valid and reliable measurement tool that can be used in Turkish society to assess parents’ attitudes toward their preschool children’s nutrition, physical activity, sedentary behaviors, and sleep. To our knowledge, the Turkish adaptation of the PRELSA scale is one of the first comprehensive measurement tools to jointly address parental attitudes toward children’s nutrition and lifestyle in the preschool period.

In the present study, the Turkish adaptation of the scale underwent a comprehensive evaluation of both linguistic and content validity. The forward–backward translation procedures demonstrated that the core conceptual framework and semantic consistency of the original instrument were largely maintained, while the items were appropriately tailored to the cultural context. Expert feedback was examined using the Davis method to quantify content validity. I-CVI and S-CVI indices both exceeded the commonly accepted cutoff value of 0.80 [19, 23]. These results indicate that the items adequately represent the targeted construct within the Turkish cultural context and demonstrate a high degree of agreement among experts. Furthermore, the content validity outcomes are consistent with the criteria established in the international literature [19, 23, 24].

Construct validity represents a core criterion in determining the extent to which a measurement instrument reliably and accurately captures the intended construct [25]. Exploratory Factor Analysis (EFA) and Confirmatory Factor Analysis (CFA) are among the most widely applied statistical techniques for evaluating this aspect of validity [26]. Before proceeding with factor analysis, it is necessary to examine the KMO coefficient and Bartlett’s sphericity test. Values exceeding 0.60 for KMO and a significant Bartlett’s result indicate that the sample provides adequate conditions for factor extraction [27]. The KMO value obtained in this study is 0.719, and the significant Bartlett test result (*χ²*=3505.367, *p* < 0.001) strongly supports the suitability of the data for factor analysis. Similarly, in the original PRELSA Scale study developed by Carretero-Bravo et al., the KMO value of 83% and the Bartlett result being found to be significant demonstrate that the Turkish version is based on reliable methodological foundations. As a result of the EFA, it was determined that the factor loadings supporting the multidimensional structure of the scale ranged from 0.353 to 0.890 and explained 58.5% of the total variance. The literature suggests that factor loadings should be at least 0.30, and that explaining 40–60% of the variance is sufficient [19, 24]. The original study reported that the scale explained 61.3% of the total variance [16]. In comparison, the Turkish version demonstrated a similar level of explained variance, indicating that parental attitudes toward nutrition, lifestyle, and sleep may be interpreted within a shared cross-cultural factor framework.

In the Turkish adaptation of the PRELSA Scale, the structure evolved from a 48-item, 14-factor model to a 39-item, 13-factor model following item removal and factor restructuring. A more detailed examination of these modifications is warranted. Regarding item removal, six items (6, 10, 12, 13, 14, and 37) yielded factor loadings below 0.30, and three items (8, 23, and 24) exhibited problematic cross-loadings. These findings suggest that some constructs represented by the removed items may not have been interpreted as distinct dimensions within the Turkish sample. Such differences are frequently reported in cross-cultural adaptation studies and may reflect variations in cultural norms, parenting practices, and perceptions related to child feeding and lifestyle behaviors [28–30]. A notable structural change was the emergence of a new factor, “Family-Based Feeding Expectations (FBFE),” formed by the co-loading of Item 11 (originally in Pressure to Eat) and Item 25 (originally in Family Meal Environment). Conceptually, both items relate to parental expectations regarding children’s eating behaviors within the family context. Their clustering may indicate that these behaviors are perceived as closely related aspects of feeding practices in the Turkish sample rather than as separate constructs. Additionally, sleep-related items 44 and 45 loaded onto the screen-use factor rather than the original sleep modification subscale. One possible explanation is that parents may perceive screen exposure and sleep habits as interconnected lifestyle behaviors. This interpretation is consistent with previous research reporting associations between screen use and sleep-related outcomes in young children [9, 10]. These structural differences should be considered when interpreting cross-cultural findings, as they may limit the direct comparability of subscale scores between the Turkish and Spanish versions of the PRELSA Scale due to differences in both item composition and dimensional organization. Although deviations from the original structure are commonly reported in cross-cultural adaptation research and do not necessarily compromise validity [28–31], the extent of the modifications observed in the present study suggests that the Turkish version should be considered a culturally adapted instrument derived from the original PRELSA framework rather than a direct structural replication of the Spanish version. CFA findings confirmed the multi-factor structure of the adapted scale. The χ²/df ratio of 1.434 (< 5, indicating acceptable fit) [19], an RMSEA value of 0.031 (< 0.08), an SRMR value of 0.046 (< 0.08), and CFI, IFI, GFI, and TLI values above 0.90 indicate that the model demonstrated acceptable fit [32, 33]. These results demonstrate that the adapted 39-item, 13-factor structure can be meaningfully and validly represented within the Turkish cultural context. It is also noteworthy that the presence of two-item factors is not unique to the Turkish adaptation; the original Spanish PRELSA Scale likewise retained five factors represented by only two items following psychometric refinement. The developers acknowledged this issue as a structural limitation and suggested that future studies explore higher-order factor models to improve interpretability and address the limited number of indicators within some dimensions [16].

In this study, the internal consistency of the PRELSA Scale was demonstrated by a Cronbach’s alpha value of 0.887. Similarly, Carretero-Bravo et al., reported a Cronbach’s alpha value of 0.89 for the original scale. Cronbach’s alpha, as a measure of internal consistency, indicates how well the items function together to assess a common underlying construct. The coefficient obtained in this study, falling between 0.80 and 1.00, suggests a high level of internal consistency [32–36]. These results indicate that the scale has good internal consistency, is reliable, adequately covers the relevant topic, and that there is consistency among the items [37–39].

The positive and significant correlations observed among the subscales indicate that parental attitudes toward their preschool children’s eating, lifestyle, and sleep behaviors are interrelated and collectively reflect an overarching construct. The correlation coefficients ranging from low to moderate levels indicate that the subscales share a common overarching conceptual framework, while each dimension represents a distinct and distinguishable construct [19, 40]. The literature indicates that a total-item correlation coefficient of 0.20 or above is acceptable, particularly in terms of the discriminative power of items in multidimensional scales [19, 41]. In this regard, it can be said that the items in the Turkish version of the PRELSA Scale generally demonstrate sufficient internal consistency. This finding supports the multidimensional theoretical structure of the PRELSA Scale.

In this study, test-retest reliability analysis was performed to evaluate the temporal stability of the scale and its consistency in repeated measurements. The test–retest results revealed a strong and statistically significant correlation between the scores obtained at the two measurement points (*r* = 0.911, *p* < 0.001) The test-retest reliability coefficient of 0.911 obtained for the total scale is above the threshold value of 0.90 recommended in the literature, indicating an excellent level of temporal stability [42–44]. The fact that the test-retest reliability coefficients at the subscale level range from 0.730 to 0.902 indicates that all subscales demonstrate acceptable levels of temporal consistency [43]. The ICC value of 0.865 (95% CI: 0.738–0.933) indicates good temporal stability, consistent with commonly accepted criteria. This finding is further supported by the high test–retest correlation (*r* = 0.911, *p* < 0.001). Together, these indices provide strong and converging evidence for the temporal reliability of the Turkish PRELSA Scale. These findings demonstrate that the Turkish version of the PRELSA Scale not only provides immediate internal consistency but also produces consistent results over time, thereby strengthening the evidence regarding the reliability of the measurement tool [33, 40, 45].

## Strengths and limitations

A major strength of this study is the rigorous cross-cultural adaptation of the PRELSA Scale using established psychometric procedures. The combined use of exploratory and confirmatory factor analyses provided robust evidence for construct validity while preserving the core theoretical framework. The multidimensional structure allows assessment of parental obesity-related attitudes across eating practices, physical activity, screen use, and sleep routines, addressing a gap in validated instruments for preschool populations in Türkiye. Test–retest analysis further supported temporal stability. Several limitations should be noted. The sample was drawn exclusively from a single geographic region (Central Anatolia), which may limit the generalizability of the findings to other regions of Türkiye. In addition, the sociodemographic profile of the participants was relatively homogeneous, residing in urban areas, and reporting similar income and educational levels. Therefore, the sample may not fully reflect the socioeconomic and cultural diversity of the broader Turkish population. Future studies should examine the psychometric properties of the scale in more geographically, socioeconomically, and culturally diverse samples. A further limitation of this study is that both the EFA and CFA were conducted using the same sample of 460 participants. This approach was necessitated by recruitment constraints, as data were collected from seven kindergartens within a single geographical region, making it difficult to obtain an independent validation sample. Although the use of a single sample for both analyses has been reported in cross-cultural adaptation studies under similar conditions, it may lead to overestimation of model fit indices and limits the independence of the validation process. Consequently, the CFA does not constitute a fully independent test of the factor structure identified through EFA, which may restrict the generalizability of the structural validity findings. Future research should therefore seek to replicate the factor structure in larger, independent, and more diverse samples. Although the Turkish adaptation involved item reduction and structural modifications that are common in cross-cultural adaptation research, it should be noted that five of the thirteen subscales consist of only two items (PRE, SMT, MOT, FBFE, and IRS). While this characteristic is also present in the original Spanish version of the PRELSA Scale [16], two-item factors are generally considered weakly identified in CFA models and may limit the stability and interpretability of the factor structure. Future studies should explore second-order factor structures or consider adding items to strengthen the psychometric identification of these subscales. Finally, the majority of participants were mothers, which limits the representativeness of the findings with respect to fathers. As paternal attitudes toward child feeding, physical activity, and sleep behaviors may differ from those of mothers, future studies should seek to recruit more gender-balanced samples to ensure broader applicability of the scale across parental roles.

## Conclusions

The psychometric findings indicate that the Turkish adaptation of the PRELSA Scale demonstrates satisfactory validity and reliability for assessing parental attitudes toward preschool obesity (2–6 years). The multidimensional structure addressing eating habits, physical activity, screen use, and sleep routines was largely preserved despite item reduction and partial factor modifications, reflecting expected cultural differences in cross-cultural adaptation studies. The 39-item, 13-subscale structure provides a culturally appropriate tool for systematically assessing parental obesity-related attitudes in Türkiye. Higher total scores indicate lower levels of risk-related attitudes. The scale may facilitate early identification of modifiable parental risk factors and support the development of targeted, family-centered interventions. Future studies should examine associations with children’s anthropometric indicators and evaluate applicability across diverse socioeconomic groups.

## Supplementary Information

Supplementary Material 1.

## Data Availability

The data that support the findings of this study are available from the corresponding author upon reasonable request.

## Abbreviations

PRELSA: Preschool eating, lifestyle, and sleeping attitudes
ICC: Intraclass correlation coefficients
WHO: World Health Organization
EFA: Exploratory Factor Analysis
CFA: Confirmatory Factor Analysis
KMO: Kaiser–Meyer–Olkin
χ²/df: Chi-square to degrees of freedom ratio
RMSEA: Root Mean Square Error of Approximation
SRMR: Standardized Root Mean Square Residual
CFI: Comparative Fit Index
IFI: Incremental Fit Index
GFI: Goodness-of-Fit Index
TLI: Tucker–Lewis Index
