# Supplementary material for: Psychometric properties of the Turkish version of the Preschool Eating, Lifestyle, and Sleeping Attitudes (PRELSA) scale
Source: BMC Pediatr. 2026 Aug 14;26:847. doi: 10.1186/s12887-026-07532-9 (PMC13560222; doi:10.1186/s12887-026-07532-9)
Supplement: Supplementary file 1 [file 12887_2026_7532_MOESM1_ESM.docx]

**APPENDIX A**

**Supplementary File 1. Preschool Eating, Lifestyle, and Sleeping Attitudes (PRELSA) Scale**

| Dear Parent;  Please answer the first 2 questions as follows: “1=Not worried at all, 2=A little worried, 3=Worried, 4=Quite worried, 5=Very worried.” For all other questions, please select the statement that best applies to you: “1=Disagree, 2=Slightly disagree, 3=Indifferent/don’t care, 4=Slightly agree, 5=Agree.” | Disagree | Slightly disagree | Indifferent/  don’t care | Slightly agree | Agree |
| --- | --- | --- | --- | --- | --- |
| ***1.** Are you worried that your child eats too much? |  |  |  |  |  |
| ***2.** Are you concerned that potential excess weight in your child during  this preschool stage could be a health problem in the short/medium term? |  |  |  |  |  |
| ***3.** I consider it appropriate to use some type of food as a reward when  behaving well. |  |  |  |  |  |
| ***4.** I consider it appropriate to use food to calm my child down. |  |  |  |  |  |
| ***5.** I consider it appropriate to offer rewards to my child to keep them still. |  |  |  |  |  |
| ***7.** I believe my child should always finish all the food on the plate. |  |  |  |  |  |
| ***9.** I try to make my child eat regardless of whether they say they are not  hungry. |  |  |  |  |  |
| ***11.** I consider it necessary for my child to eat enough, even if the diet is  sometimes not varied. |  |  |  |  |  |
| **15.** I consider it necessary to always offer salads and/or vegetables in my  child's meals. |  |  |  |  |  |
| **16.** I consider it necessary for my child to eat fruits every day. |  |  |  |  |  |
| **17.** I consider it necessary for family meals to always have dishes with  salads and/or vegetables on the table. |  |  |  |  |  |
| ***18.** I consider it appropriate for my child to get up or move around during  meals. |  |  |  |  |  |
| **19.** I consider it appropriate for my child to remain seated throughout  mealtime. |  |  |  |  |  |
| ***20.** I consider it appropriate for my child to eat if they are not seated at the  table. |  |  |  |  |  |
| **21.** I consider it appropriate for my child to be able to decide when he/she  wants to eat. |  |  |  |  |  |
| **22.** I consider it appropriate for my child to sometimes eat between routine  meals. |  |  |  |  |  |
| **25.** I consider it necessary for my child to eat the same food as the rest of the family. |  |  |  |  |  |
| ***26.** I consider it appropriate for my child to eat entertained with screens  (TV, computer, mobiles, and/or tablets). |  |  |  |  |  |
| ***27.** I consider it appropriate for my child to eat entertained with some game. |  |  |  |  |  |
| ***28.** I consider it appropriate to use screens during meals. |  |  |  |  |  |
| **29.** I consider my child's physical activity as important for their development as their nutrition. |  |  |  |  |  |
| **30.** I consider my child's physical activity as important for their development as their studies. |  |  |  |  |  |
| **31.** I try to teach my child that being active is good for their health. |  |  |  |  |  |
| **32.** I consider it important to introduce my child to various options for  physical activity and active play. |  |  |  |  |  |
| **33.** I believe my child can learn healthy habits of physical activity through  my example. |  |  |  |  |  |
| **34.** I consider it necessary to provide my child with toys and/or games that  encourage physical activity. |  |  |  |  |  |
| **35.** I consider it necessary to organize my child's routines so that there is  frequently space for physical activity. |  |  |  |  |  |
| **36.** One of my priorities when thinking about where to live is that my child  has spaces to play indoors or outdoors. |  |  |  |  |  |
| **38.** I believe it's necessary to have time for physical activity with my child. |  |  |  |  |  |
| **39.** I believe family habits of physical activity can influence my child's  healthy lifestyle throughout their life. |  |  |  |  |  |
| **40.** I consider it appropriate to motivate my child to engage in physical  activities over other types of activities. |  |  |  |  |  |
| **41.** I consider it appropriate to play active games and/or sports with my  child over other activities. |  |  |  |  |  |
| **42.** I consider it appropriate to limit my child's screen viewing (TV, computer, mobiles, and/or tablets). |  |  |  |  |  |
| **43.** I consider it appropriate to limit my screen time when I'm with my  child/children. |  |  |  |  |  |
| **44.** I believe sleep routines affect other areas of my child's mental and  physical development. |  |  |  |  |  |
| **45.** I believe my child's lack of sleep can encourage them to eat more during  the day. |  |  |  |  |  |
| **46.** I believe family routines can influence proper sleep patterns for children. |  |  |  |  |  |
| **47.** I consider it appropriate for my child to have the same sleep habits every  day of the week. |  |  |  |  |  |
| **48.** I consider it appropriate for my child to sleep most nights in the same  place. |  |  |  |  |  |

**Scale Description:** To assess the construct validity of the scale, exploratory factor analysis (EFA) was first applied, followed by confirmatory factor analysis (CFA) to validate the obtained structure. As a result of the analyses, factor loadings of the 48-item PRELSA scale were examined, and six items (6, 10, 12, 13, 14, and 37) with factor loadings below 0.30 were removed from the scale because they did not adequately represent the relevant factor. In addition, three items (8, 23, and 24) with cross-loading differences below 0.10 and similar loadings on more than one factor were considered problematic and excluded from the analysis. Following these modifications, the scale reached a final structure consisting of 39 items and 13 factors. The sub-factors are named Parental Concern about the Child’s Weight (CWC) (1, 2), Use of Food as a Reward (REW) (3, 4, 5), Pressure to Eat (PRE) (7, 9), Variety and Quality of the Diet (VQD) (15, 16, 17), Movement during Mealtime (MOV) (18, 19, 20), Structured Mealtime (SMT) (21, 22), Family-Based Feeding Expectations (FBFE) (11, 25), Use of Distractions during Mealtime (DIS) (26, 27, 28), Importance of Physical Activity (IPA) (29, 30, 31, 38, 39), Parental Support and Co-Participation in Physical Activity (SCP) (32, 33, 34, 35, 36), Motivation to Physical Activity (MOT) (40, 41), Attitudes Toward Screen Use and Sleep Patterns (ATSP) (42, 43, 44, 45) and Importance of Routines regarding Sleep (IRS) (46, 47, 48) in order. Items numbered 1, 2, 3, 4, 5, 7, 9, 11, 18, 20, 26, 27, and 28 on the scale were reverse-coded for analysis.
